# Safety of denervation following targeted lung denervation therapy for COPD: AIRFLOW-1 3-year outcomes

**DOI:** 10.1186/s12931-021-01664-5

**Published:** 2021-02-19

**Authors:** Pison Christophe, L. Shah Pallav, Slebos Dirk-Jan, Ninane Vincent, Janssens Wim, Perez Thierry, Kessler Romain, Deslee Gaetan, L. Garner Justin, E. Hartman Jorine, Degano Bruno, Mayr Anna, Mayse Martin, D. Peterson Alexander, Valipour Arschang

**Affiliations:** 1 Service Hospitalier Universitaire Pneumologie Physiologie, Pôle Thorax et Vaisseaux, CHU Grenoble Alpes, CS10217, 38043 Grenoble Cedex 9, France; 2 Université Grenoble Alpes, Grenoble, France; 3 Royal Brompton & Harefield NHS Trust, Chelsea & Westminster Hospital and Imperial College, London, UK; 4 Department of Pulmonary Diseases, University of Groningen, University Medical Center Groningen, Groningen, The Netherlands; 5 CHU Saint-Pierre, Université Libre de Bruxelles, Brussels, Belgium; 6 Department of Respiratory Diseases, KU Leuven, University Hospitals Leuven, Leuven, Belgium; 7 CHU Lille, Center for Infection and Immunity of Lille, INSERM U1019-UMR9017, Univ Lille Nord de France, Lille, France; 8 Service de Pneumologie, Nouvel Hôpital Civil, Université de Strasbourg, Strasbourg, France; 9 Service de Pneumologie, INSERM UMRS-1250, CHU de Reims, Hôpital Maison Blanche, Reims, France; 10 Department of Respiratory and Critical Care Medicine, Karl-Landsteiner-Institute for Lung Research and Pulmonary Oncology, Klinik Floridsdorf, Vienna, Austria; 11 Nuvaira, Inc., Minneapolis, MN USA

**Keywords:** COPD, Nerves, Targeted lung denervation, Acetylcholine, Anticholinergic, Bronchoscopy

## Abstract

**Background:**

Targeted lung denervation (TLD) is a novel bronchoscopic therapy that disrupts parasympathetic pulmonary nerve input to the lung reducing clinical consequences of cholinergic hyperactivity. The AIRFLOW-1 study assessed safety and TLD dose in patients with moderate-to-severe, symptomatic COPD. This analysis evaluated the long-term impact of TLD on COPD exacerbations, pulmonary function, and quality of life over 3 years of follow up.

**Methods:**

TLD was performed in a prospective, energy-level randomized (29 W vs 32 W power), multicenter study (NCT02058459). Additional patients were enrolled in an open label confirmation phase to confirm improved gastrointestinal safety after procedural modifications. Durability of TLD was evaluated at 1, 2, and 3 years post-treatment and assessed through analysis of COPD exacerbations, pulmonary lung function, and quality of life.

**Results:**

Three-year follow-up data were available for 73.9% of patients (n = 34). The annualized rate of moderate to severe COPD exacerbations remained stable over the duration of the study. Lung function (FEV_1_, FVC, RV, and TLC) and quality of life (SGRQ-C and CAT) remained stable over 3 years of follow-up. No new gastrointestinal adverse events and no unexpected serious adverse events were observed.

**Conclusion:**

TLD in COPD patients demonstrated a positive safety profile out to 3 years, with no late-onset serious adverse events related to denervation therapy. Clinical stability in lung function, quality of life, and exacerbations were observed in TLD treated patients over 3 years of follow up.

## Background

COPD is characterized by persistent limitation of airflow and respiratory symptoms resulting from abnormalities in the lungs at the level of the alveoli, airways, or both [1, 2]. COPD exacerbations are associated with high costs, poor clinical prognosis, increased morbidity and increased mortality; therefore, reducing the risk of exacerbations in the COPD population is an important treatment goal [1, 3, 4].

The pathophysiology of COPD is characterized by increased autonomic nervous system input to the lung and sensory signaling from the lung mediated by the vagus nerve. Increased parasympathetic input raises cholinergic tone in the airways which modulates airway smooth muscle tone, airway hyperresponsiveness, inflammation and mucus hypersecretion [5–7]. Thus, one of the cornerstones of COPD pharmaceutical management is to reduce symptoms by blocking cholinergic signaling with anticholinergic agents. Anticholinergic agents relax airway smooth muscle, decrease mucus hypersecretion, and improve COPD-related symptoms [1, 8].

Medical therapy has limitations and leaves significant unmet needs for COPD patients [1, 9–11]. Patient compliance to prescribed inhaled medications and optimal deposition of medication into the lung periphery remain challenges [12]. Acute changes in lung physiology due to infections/inflammation overwhelm the therapeutic effect of inhaled medications [13]. As a result, patients continue to exacerbate while on medical therapy, with only about 50% of patients treated with a long acting muscarinic antagonist experiencing a clinically significant benefit [14] and up to 70% of patients experiencing a COPD exacerbation while on optimal medical therapy [11].

Targeted lung denervation (TLD) is a novel bronchoscopic therapy that interrupts parasympathetic pulmonary nerve input to the lung to reduce clinical consequences of cholinergic hyperactivity [15–17]. Previous research of TLD has established an optimal dosing strategy and the safety of the procedure [16, 18]. This study presents the long-term impact of TLD on COPD exacerbations, symptoms, quality of life and pulmonary function at 3 years post-TLD as observed in the AIRFLOW-1 clinical trial.

## Methods

Briefly, TLD therapy is delivered via a dual-cooled radiofrequency (RF) catheter (Nuvaira, Minneapolis, Minnesota, USA) designed to target tissue heating at depth, thereby producing a narrow band of ablation around the main bronchi while minimizing effects to the inner surface of the airway. As RF current passes from the electrode through the airway and surrounding tissues, these tissues are heated. Coolant continuously circulated through the electrode and balloon removes heat from the surface of the airway wall. The net effect is targeted tissue ablation at depth with minimal heating and damage of the inner surface of the airway [15–17].

AIRFLOW-1 was a randomized (1:1) double-blind multicenter study (NCT02058459) of 30 patients with moderate-to-severe symptomatic COPD that underwent TLD at 29 W (n = 15) or 32 W (n = 15) power. An additional 16 patients were treated with 32 W power, associated with optimal improvements in FEV_1_ and SGRQ-C, after randomization as an open-label group to confirm safety improvements after procedural enhancements following gastrointestinal adverse events during the randomized part of the trial [16]. Detailed descriptions of the study methodology have been previously published [16]. All patients signed a written informed consent form, and study approval was obtained from local ethics committees and in accordance with the Declaration of Helsinki (1996), Good Clinical Practice guidelines, and any local requirements.

Gastrointestinal events were reported during the randomization phase, which led to procedural modifications and a subsequent reduction in treatment-related gastric side-effects in the open-label phase [16]. The procedural change was the addition of esophageal balloon placement allowing the physician to visualize the esophagus and fluoroscopically measure the distance between the radiofrequency (RF) electrode and the esophagus. Additionally, a treatment plan was developed to lower the energy dose depending on proximity to the esophagus.

### Outcome measures

**Fig. 1:**
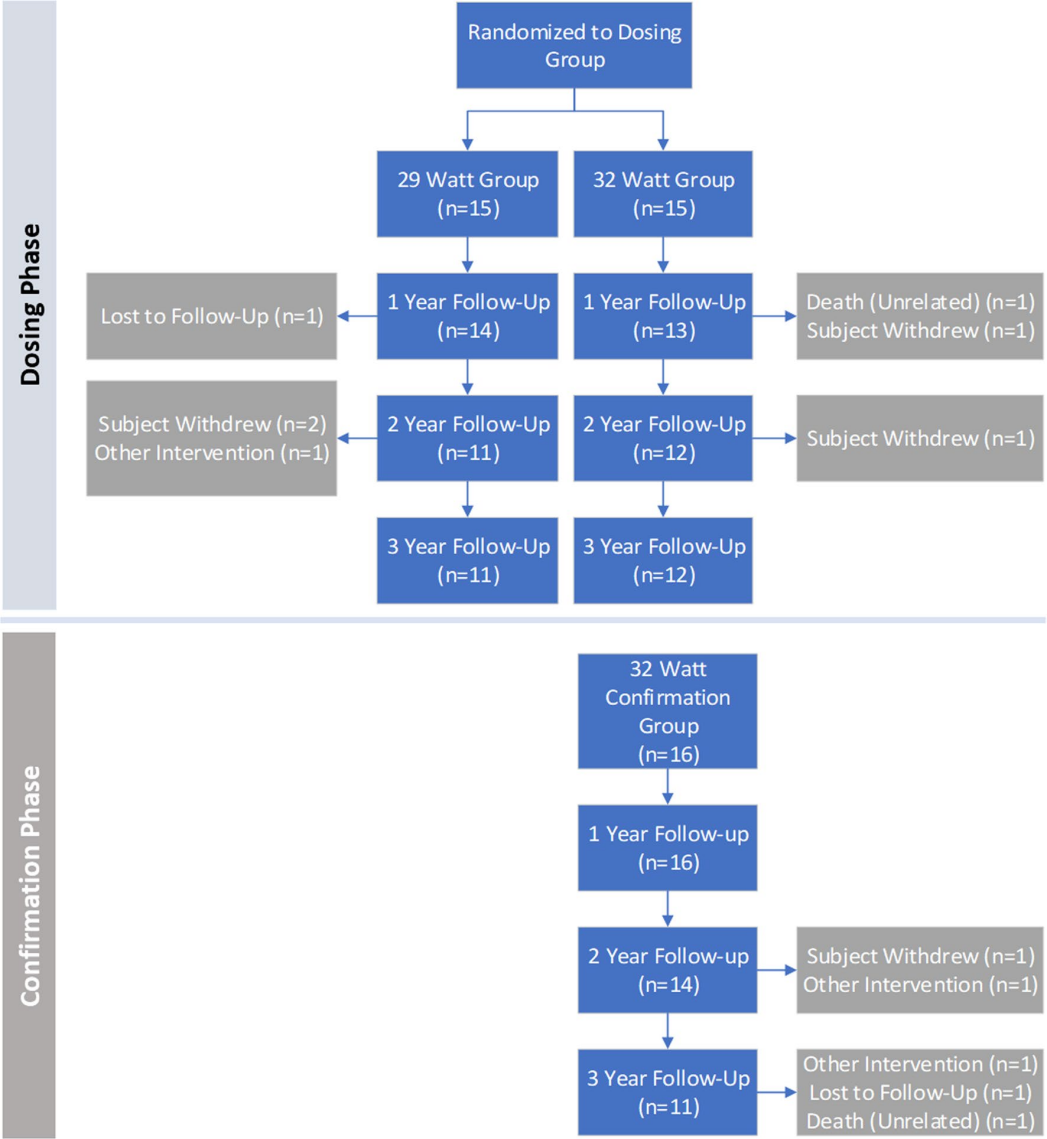
Participant flow through the study. Dosing phase for the 29 W and 32 W groups and confirmation phase for the unblinded 32 W confirmation group

TLD’s impact on COPD exacerbations (moderate to severe) along with pulmonary lung function and health-related quality of life was evaluated at 1, 2, and 3 years post-treatment. Patient flow through the study from baseline through the 3-year follow-up is described in Fig. 1. Washout measures of pulmonary lung function evaluated were FEV_1_, FVC, RV, and TLC and were compared with baseline at 1, 2, and 3 years post-treatment. Health-related quality of life was measured with the COPD-specific St. George’s Respiratory questionnaire (SGRQ-C) and COPD Assessment Test (CAT) and compared with baseline at 1, 2, and 3 years post-TLD.

### Statistical analysis

Statistical hypothesis tests were performed on all subjects (n = 46) combined into one group as sample size of individual groups was small. Tests were based on paired t-tests for repeated and continuous data that are normally distributed. Non-parametric tests were performed when there was evidence of non-normality. Data at 1, 2, and 3-year time points were compared against baseline and P < 0.05 was considered statistically significant.

## Results

A total of 46 patients were treated with TLD during the initial study enrollment period; baseline characteristics have been previously described [16]. Retention was high with 80.4% follow-up at 2 years and 73.9% follow-up at 3 years (Fig. 1).

### Safety

Over the 2nd and 3rd years of follow-up, there were no new gastrointestinal serious adverse events related to the procedure or device (SAEs; Table 1). A patient in the confirmation group experienced a gastric SAE at 650 days post procedure. While the primary gastrointestinal events experienced during the randomization phase were impaired gastric emptying, this patient experienced increased dysphagia which fully resolved and was determined not to be related to the procedure or the device. There was one death in the confirmation group 1128 days post procedure (Fig. 1). The acute cardiac death was determined unrelated to TLD and no autopsy was performed. No unanticipated SAEs were seen 3 years out from the TLD procedure in lung denervated patients (Table 1).

**Table 1:** Serious adverse events

Group (# subjects)	All-cause			Respiratory			Gastrointestinal			Cardiac		
	Year 1	Year 2	Year 3	Year 1	Year 2	Year 3	Year 1	Year 2	Year 3	Year 1	Year 2	Year 3
29 W (n = 15)	9 (16)	1 (1)	5 (9)	3 (5)	1 (1)	5 (8)	6 (7)	0 (0)	0 (0)	0 (0)	0 (0)	0 (0)
32 W (n = 15)	5 (14)	1 (1)	5 (9)	3 (5)	0 (0)	4 (6)	3 (4)	0 (0)	0 (0)	2 (2)	1 (1)	1 (1)
32 W-CFM (n = 16)	5 (9)	6 (9)	6 (8)	4 (4)	6 (6)	5 (6)	0 (0)	1 (1)	0 (0)	0 (0)	1 (1)	2 (2)

Number of patients with a serious adverse event within the 29 W, 32 W and 32 W confirmation (32 W-CFM) groups. Values are # of patients, (# of events)

**Fig. 2:**
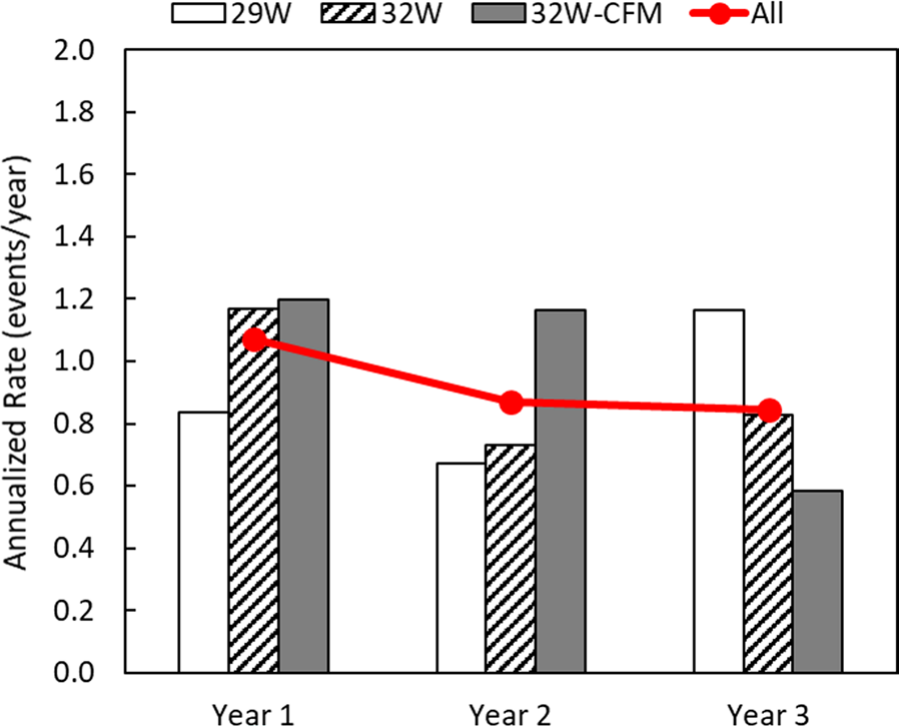
Annualized moderate to severe COPD exacerbation rate. Annualized rate for the 29 W group (open bars), 32 W group (diagonal bars) and confirmation group (32 W-CFM; gray bars). Combined groups, “All”, are depicted as the red line

The percentage of patients having at least one moderate or severe COPD exacerbation was 70% (31/44), 61% (23/38), and 46% (16/35) at the 1, 2 and 3-year follow-up respectively. Annualized rate of moderate or severe COPD exacerbations remained stable over the duration of the study (Fig. 2).

### Pulmonary function and quality of life

**Fig. 3:**
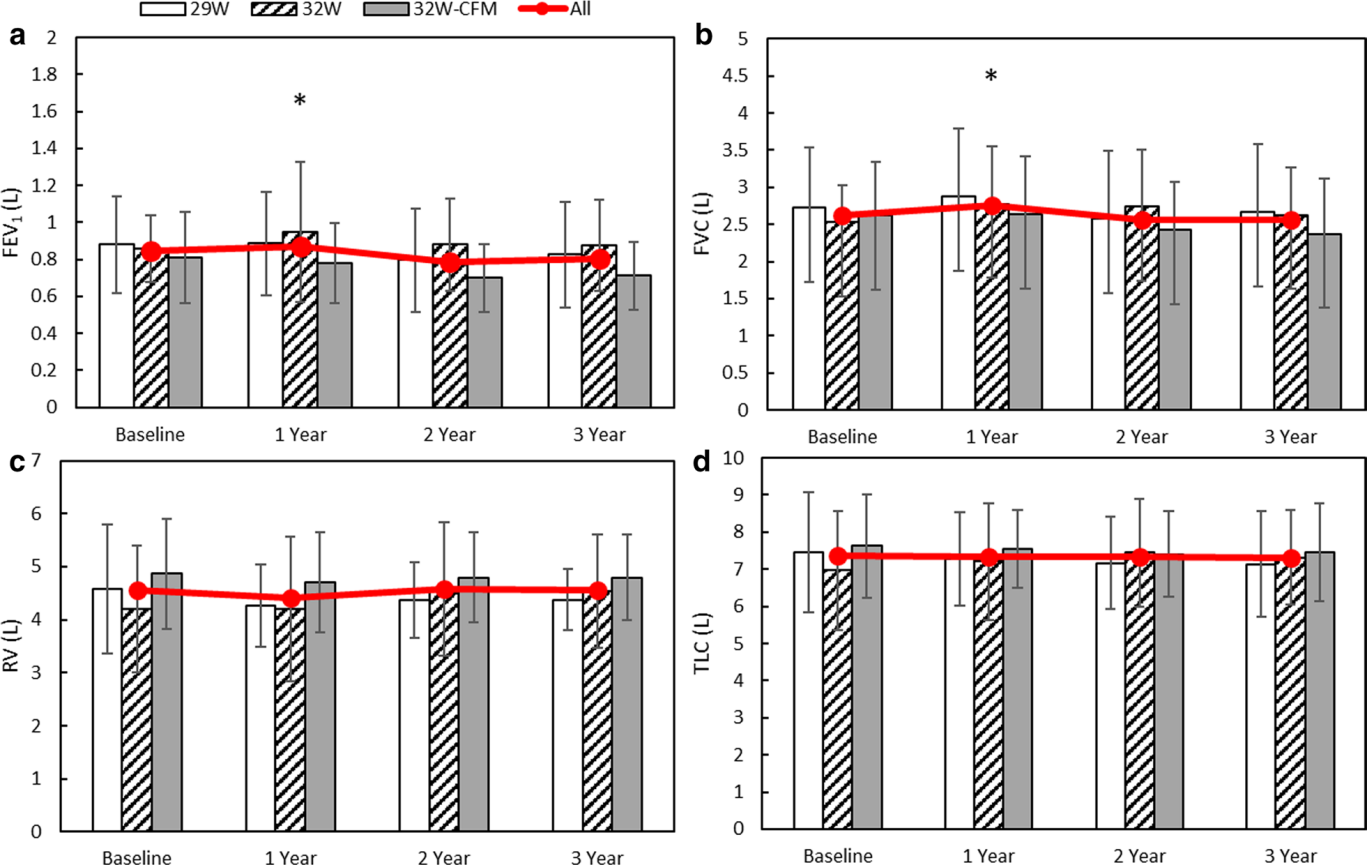
Long-term lung function. **a** FEV_1_, **b**, FVC, **c** RV, and **d** TLC for the 29 W group (open bar), 32 W group (diagonal line bar) and 32 W confirmation group (32 W-CFM, gray bar) collected at baseline, 1, 2, and 3 years after TLD. Data presented are means ± SD. Combined values, “All”, are depicted as the red line. P < 0.05 for combined values compared with baseline is denoted by *. *FEV*_*1*_ forced expiratory volume in 1 s, *FVC* forced vital capacity, *RV* residual volume, *TLC* total lung capacity, *TLD* targeted lung denervation

**Fig. 4:**
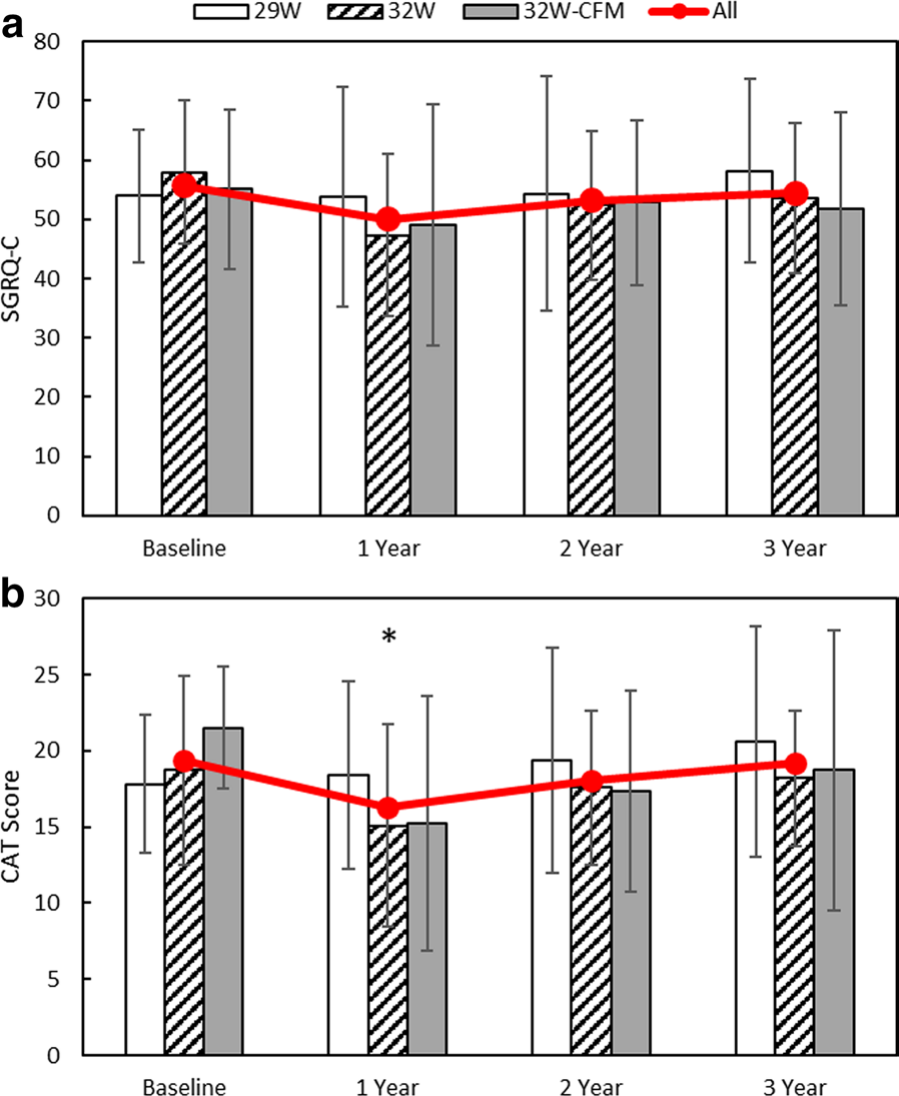
Quality of life assessment. **a** Saint George’s Respiratory Questionnaire (SGRQ-C) and **b** COPD assessment test (CAT) for the 29 W group (Open Bars), 32 W Group (hashed bars), and 32 W confirmation group (32 W-CFM; solid gray bars) at baseline, 1, 2, and 3 years. Values are mean ± SD. Combined values, “All”, are depicted as the red line. P < 0.05 for combined values compared with baseline is denoted by *

Both FEV_1_ and FVC improved at 1 year relative to baseline with average increases of 60 mL (P = 0.031) and 219 mL (P = 0.004) respectively. These measures were not significantly different from baseline at the 2 and 3-year follow-up visits. Figure 3 shows that overall lung function (FEV_1_, FVC, RV, and TLC) was stable during the 3 years of follow-up. CAT also improved at 1 year relative to baseline with an average decrease of − 3.03 points (P = 0.032) but was not significantly different at the 2 or 3-year follow-up visits. Figure 4 shows that overall quality of life and symptoms (SGRQ and CAT) were stable during the 3 years of follow up.

## Discussion

These data add to previously published research on the safety and feasibility of the TLD procedure [15–18]. Three years of follow-up demonstrate a positive long-term safety profile, and no new GI-related SAEs related to the procedure or device over the 2nd and 3rd year of follow up. Patients treated with TLD had stable lung function and symptom levels at 3 years post treatment.

A previously published TLD clinical study using an earlier version of the device not yet compatible with flexible bronchoscopy also followed subjects for 3 years of follow-up [17]. Both long-term studies utilizing different generation devices and associated doses yielded results supporting favorable long-term safety. In addition, the absence of unexpected severe adverse events in the confirmation group indicates that the procedural modifications to fluoroscopically measure distance from the esophagus did not result in any unanticipated adverse events during later years [16].

It is well established that lung function in COPD patients continues to decline with progression of the disease. Prior longitudinal studies across a wide range of GOLD stage COPD patients has consistently reported an average annual rate of decline in FEV_1_ and FVC to be ~ 30 mL/year and ~ 40 mL/year respectively [19, 20]. Annual decline in FEV_1_ specific to GOLD Stage II and III COPD patients has been reported to range from ~ 40–80 mL/year and ~ 30–60 mL/year respectively [21, 22]. Similarly, decline in patient reported quality of life measures have been reported in COPD patients with an average yearly increase in SGRQ-C of 1.2 to 1.8 points being reported [19, 23].

Previous TLD clinical studies have demonstrated potential positive impacts on lung function and quality of life measures in patients with COPD [15–18]. The current results suggest that these impacts on lung function and COPD symptoms remained largely stable over the 3 years of follow up. At 3 years post-treatment, measures of lung function (FEV_1_, FVC, RV, and TLC) and quality of life (SQRG-C and CAT) on average were similar to baseline measures and absent of significant declines.

Follow-up bronchoscopy was performed at 3 months post-treatment checking for presence of adverse reactions within the airways such as narrowing, stenosis, fibrosis, etc. Although no follow-up bronchoscopy was performed at later time points, stability of lung function may also support the absence of long-term adverse airway reactions occurring beyond 3 months post-treatment.

The current study has limitations, principally the small cohort size, and a lack of a sham-control group. However, a strength of this study is its high retention through 3 years of follow-up, which is rare in early-stage device trials. Moreover, any placebo effect assumed by the open-label design would not be expected to impact 2 or 3-year measures. The absence of decline three years post-treatment compared with baseline supports stability for lung function and quality of life in this study and both are consistent with the stable rate of exacerbations observed in this study [24]. These observations will need to be confirmed in an ongoing large scale sham-controlled randomized trial (AIRFLOW-3) comparing the efficacy of TLD plus optimal medical care for patients with moderate-to-severe COPD against optimal medical care for COPD [25].

## Conclusion

In summary, the current study provided long-term follow-up data on TLD and confirmed a positive safety profile out to 3 years. Moderate-to-severe COPD patients treated with TLD demonstrated clinical stability in lung function, symptoms, quality of life, and exacerbations over 3 years of follow-up. No late onset gastrointestinal events or adverse events related to targeted lung denervation therapy were reported.

## Data Availability

Data are available on demand at Nuvaira, Inc., Minneapolis, MN, USA.

## Abbreviations

CAT: COPD assessment test
COPD: Chronic obstructive pulmonary disease
CFM: ConFirMation
FEV_1_: Forced expiratory volume 1 s
FVC: Forced volume capacity
GOLD: Global initiative on Obstructive Lung Disease
RV: Residual volume
SGRQ-C: Saint Georges Respiratory Questionnaire-C
TLC: Total lung capacity
TLD: Targeted lung denervation
RF: Radio frequency
SAE: Serious adverse event
W: Watt
