# Correction to: Safety of denervation following targeted lung denervation therapy for COPD: AIRFLOW-1 3-year outcomes

**DOI:** 10.1186/s12931-021-01678-z

**Published:** 2021-03-15

**Authors:** Christophe Pison, Pallav L. Shah, Dirk‑Jan Slebos, Vincent Ninane, Wim Janssens, Thierry Perez, Romain Kessler, Gaetan Deslee, Justin L. Garner, Jorine E. Hartman, Bruno Degano, Anna Mayr, Martin Mayse, Alexander D. Peterson, Arschang Valipour

**Affiliations:** 1 Service Hospitalier Universitaire Pneumologie Physiologie, Pôle Thorax et Vaisseaux, CHU Grenoble Alpes, CS10217, 38043 Grenoble Cedex 9, France; 2 Université Grenoble Alpes, Grenoble, France; 3 Royal Brompton & Harefield NHS Trust, Chelsea & Westminster Hospital and Imperial College, London, UK; 4 Department of Pulmonary Diseases, University of Groningen, University Medical Center Groningen, Groningen, The Netherlands; 5 CHU Saint-Pierre, Université Libre de Bruxelles, Brussels, Belgium; 6 Department of Respiratory Diseases, KU Leuven, University Hospitals Leuven, Leuven, Belgium; 7 CHU Lille, Center for Infection and Immunity of Lille, INSERM U1019-UMR9017, Univ Lille Nord de France, Lille, France; 8 Service de Pneumologie, Nouvel Hôpital Civil, Université de Strasbourg, Strasbourg, France; 9 Service de Pneumologie, INSERM UMRS-1250, CHU de Reims, Hôpital Maison Blanche, Reims, France; 10 Department of Respiratory and Critical Care Medicine, Karl-Landsteiner-Institute for Lung Research and Pulmonary Oncology, Klinik Floridsdorf, Vienna, Austria; 11 Nuvaira, Inc., Minneapolis, MN USA

## Correction to: Respir Res (2021) 22:62 https://doi.org/10.1186/s12931-021–01664-5

Following publication of the original article [1], we were notified that the first and last author names have been swapped.

The original article has been corrected.
